# The impact of delayed paper communication to primary care from secondary care and out of hours services

**DOI:** 10.1080/17571472.2018.1490314

**Published:** 2018-06-26

**Authors:** Dominic Crocombe, Mayukh Bhattacharyya

**Affiliations:** Barking, Havering and Redbridge University Hospitals NHS Trust, Queen’s Hospital, Romford, UK

**Keywords:** General practice, medical records, documentation, continuity of patient care

## Abstract

The safe and effective treatment of patients accessing multiple NHS services relies upon efficient communication between primary care, secondary care, and out of hours providers. There is a theoretical risk to patient safety from delays in these processes, to which paper communications are particularly vulnerable. When letters are received they must be reviewed and prioritised in order of clinical importance, a process that requires both time and clinical resources. This is relevant to the challenge of resource allocation to maximise patient benefit. This retrospective study investigated the impact on patient safety of 249 clinical letters reporting routine clinical encounters in secondary care and out of hours services that were delayed by an average of 18–24 months to a suburban London general practice. No clinical harm could be attributed to the delay. This small study did not suggest delays in routine communications pose a significant risk to patient safety. Conversely, it questions the efficiency and benefit to patients of prioritising clinical time to reviewing routine letters. The adoption of fully integrated, shared electronic patient records with the function to highlight clinically urgent or important communications might ease clinician workload, to the ultimate benefit of patient care.

## Why this matters to us

This subject is important to us because general practitioners undertake a significant administrative and bureaucratic workload. Furthermore, lost and delayed communications cause anxiety amongst clinicians, patients and management. This study adds some evidence to support a move away from prioritising the processing of routine paper communications thus allowing a redirection of clinician time and resources towards those of greater patient benefit.

## Key message

Delayed delivery of routine letters to primary care minimally impacts patient outcomes.

## Introduction

Effective communication between primary and secondary care providers is essential for patient safety and continuity of care. It is the duty of the discharging secondary care service to ensure general practitioners are kept updated in this regard [1,2]. For many decades, printed paper letters have been the standard method of communication. Despite significant and expensive efforts, the NHS has been relatively slow in adopting the rapid advances in communication technologies now ubiquitous in most modern industries. As a result, communication between most primary and secondary care providers continues to rely on paper. One of the major perceived risks with this remains physical, namely the potential to lose or misplace clinical letters to the detriment of patient care. Furthermore, when letters are received they must be reviewed and prioritised in order of clinical importance, a process that requires both time and clinical resources. This is relevant to the challenge of resource allocation to maximise patient benefit.

## Aim

Our aim was to investigate the risk to patient safety of delayed routine paper communications from secondary and out-of-hours services to primary care.

## Method

We reviewed paper communications in the form of clinical letters (*n* = 249) addressed to a large suburban general practice in east London. The letters had all been sent from secondary care and out-of-hours primary care services. All had been received late by an average of 18–24 months for various reasons including incorrect addresses and patients changing care provider. Analysis was undertaken between the dates of 6th and 23rd February 2017. Every individual letter was analysed retrospectively against the patient’s electronic medical records and electronic communications with focus on the time following the clinical encounter addressed in the delayed letter. Adverse outcomes were defined as any recorded delay or inappropriate treatment, diagnosis, referral, or other clinical complication that could reasonably be associated with or attributed to the delayed communication.

## Results

The clinical impact of most letters was minor, for example brief summaries of routine clinical encounters. There was no documented evidence that any patient experienced an adverse outcome to their safety or care that could reasonably be associated with or attributed to the delayed paper communication [Table 1]. In all cases of more serious clinical diagnoses, additional methods of communication such as electronic mail had been used to expedite the message to primary care.

**Table 1.** Results of retrospective analysis.

Delayed letters (n)	Approximate time range of delay in delivery of letters	Number of adverse outcomes to patient safety or care associated with or attributable to delayed letter
249	18–24 months	0

## Discussion

This study produced no evidence of actual adverse outcomes to patient care or safety of delayed routine clinical letters. This may provide some superficial reassurance for the continuing use of routine paper communications however the sample size was relatively small and the risk of delayed communication cannot be completely dismissed.

More intriguing was the absence of any significant clinical impact of letters delayed by long periods of time, which suggests many routine paper communications are of limited clinical value despite the time and resources required to process them. It is widely recognised that the increase in administrative and bureaucratic tasks represents a major pressure on the clinical workforce in general practice [3,4]. We would argue that routine letters from secondary and out-of-hours primary care services to general practitioners would be better delivered electronically to help reduce this burden. This should be via a shared, electronic record system with the function to highlight letters of greater clinical importance. This view is shared in previous studies investigating communication between primary care physicians and other healthcare providers [5]. Furthermore, electronic discharge summaries have been shown to improve the completeness and efficiency of communication between primary and acute hospital services [6].

Such a transition away from routine paper communications would support the current trend towards fully integrated electronic care records and communication systems between primary care and other NHS services. These are targeted at minimising cost, resource wastage, and the theoretical risk to patients of paper communication [7,8] and align with the vision shared by the Department of Health and NHS England [9]. Moreover, reducing the administrative workload of primary care clinicians would allow the redirection of time and resources into tasks of greater benefit to patients.

## Governance Information

This work was undertaken at Church Elm Lane Medical Practice, Dagenham, UK under the supervision of Dr. Mina Goyal and Dr. Alex Duodu.

## Disclosure statement

No potential conflict of interest was reported by the authors.
